# Supplementary material for: Structural and functional variation of chitin-binding domains of a lytic polysaccharide monooxygenase from Cellvibrio japonicus
Source: J Biol Chem. 2021 Aug 17;297(4):101084. doi: 10.1016/j.jbc.2021.101084 (PMC8449059; doi:10.1016/j.jbc.2021.101084)
Supplement: Supplemental Figures S1–S4 and Table S1 [file mmc1.docx]

**Structural and functional variation of chitin-binding domains of a lytic polysaccharide monooxygenase from *Cellvibrio japonicus***

Eva Madland*^1^, Zarah Forsberg*^2^, Yong Wang^3^, Kresten Lindorff-Larsen^3^, Axel Niebisch^4^, Jan Modregger^4^, Vincent G. H. Eijsink^2^, Finn L. Aachmann^1^, Gaston Courtade^1^**

^1^ Norwegian Biopolymer Laboratory (NOBIPOL), Department of Biotechnology and Food Science, NTNU Norwegian University of Science and Technology, 7491Trondheim, Norway

^2^ Faculty of Chemistry, Biotechnology and Food Science, Norwegian University of Life Sciences (NMBU), 1432 Ås, Norway

^3^ Structural Biology and NMR Laboratory, Linderstrøm-Lang Centre for Protein Science, Department of Biology, University of Copenhagen, Denmark

^4^ Eucodis Bioscience GmbH, Campus Vienna Biocenter 2, 1030 Wien, Austria

*These authors contributed equally to this work

**Corresponding author

E-mail: gaston.courtade@ntnu.no

Keywords: carbohydrate-binding domain, chitin, lytic polysaccharide monooxygenase, molecular dynamics simulations, multi-modular, nuclear magnetic resonance (NMR), substrate binding

**SUPPORTING INFORMATION**

**Table S1** Restraints and structural statistics for the 20 best conformers of the NMR solution structure of *Cj*CBM5 (PDB ID: 6Z40) and *Cj*CBM73 (PDB ID: 6Z41)

|  | | *Cj*CBM5 | *Cj*CBM73 |
| --- | --- | --- | --- |
| **Total number of NOE distance constraints** | | **435** | **656** |
|  | Intraresidue (\|i –j\|=0) | 192 | 365 |
|  | Sequential (\|i–j\|=1) | 168 | 114 |
|  | Medium-range (1<\|i–j\|<5Å) | 25 | 61 |
|  | Long-range (\|i–j\|≥5Å) | 50 | 116 |
| **Torsion angle restraints** | | **83** | **66** |
| Structure statistics (20 conformers) | |  |  |
|  | CYANA target function value (Å^2^) | 3.40 ± 0.36 | 2.83 ± 0.18 |
|  | Maximum residual distance constraint violation (Å) | 0.45 | 0.28 |
|  | Maximum torsion angle constraint violation (^o^) | 5.75 | 4.21 |
| PROCHECK-NMR Ramachandran plot analysis ^a^ | |  |  |
|  | Residues in favored regions (%) | 70.0 | 76.6 |
|  | Residues in additionally allowed regions (%) | 26.3 | 22.3 |
|  | Residues in generously allowed regions (%) | 1.1 | 1.1 |
|  | Residues in forbidden regions (%) | 1.6 | 0.0 |
| RMSD to the average coordinates (Å) | |  |  |
|  | N, C^α^, C’ ^a^ | 1.47 ± 0.27 | 1.12 ± 0.28 |
|  | Heavy atoms ^a^ | 2.00 ± 0.29 | 1.52 ± 0.39 |
|  | N, C^α^, C’ (secondary structure)^b^ | 0.45 ± 0.15 | 0.36 ± 0.11 |
|  | Heavy atoms (secondary structure)^b^ | 1.35 ± 0.20 | 0.74 ± 0.13 |

^a^ Residues used for RMSD calculation. *Cj*CBM5: 251–307; *Cj*CBM73: 338–397
^b^ Residues used for RMSD calculation. *Cj*CBM5: 271–274, 276–280, 298–301 and *Cj*CBM73: 357–359, 362–366, 371–374, 390–394

**
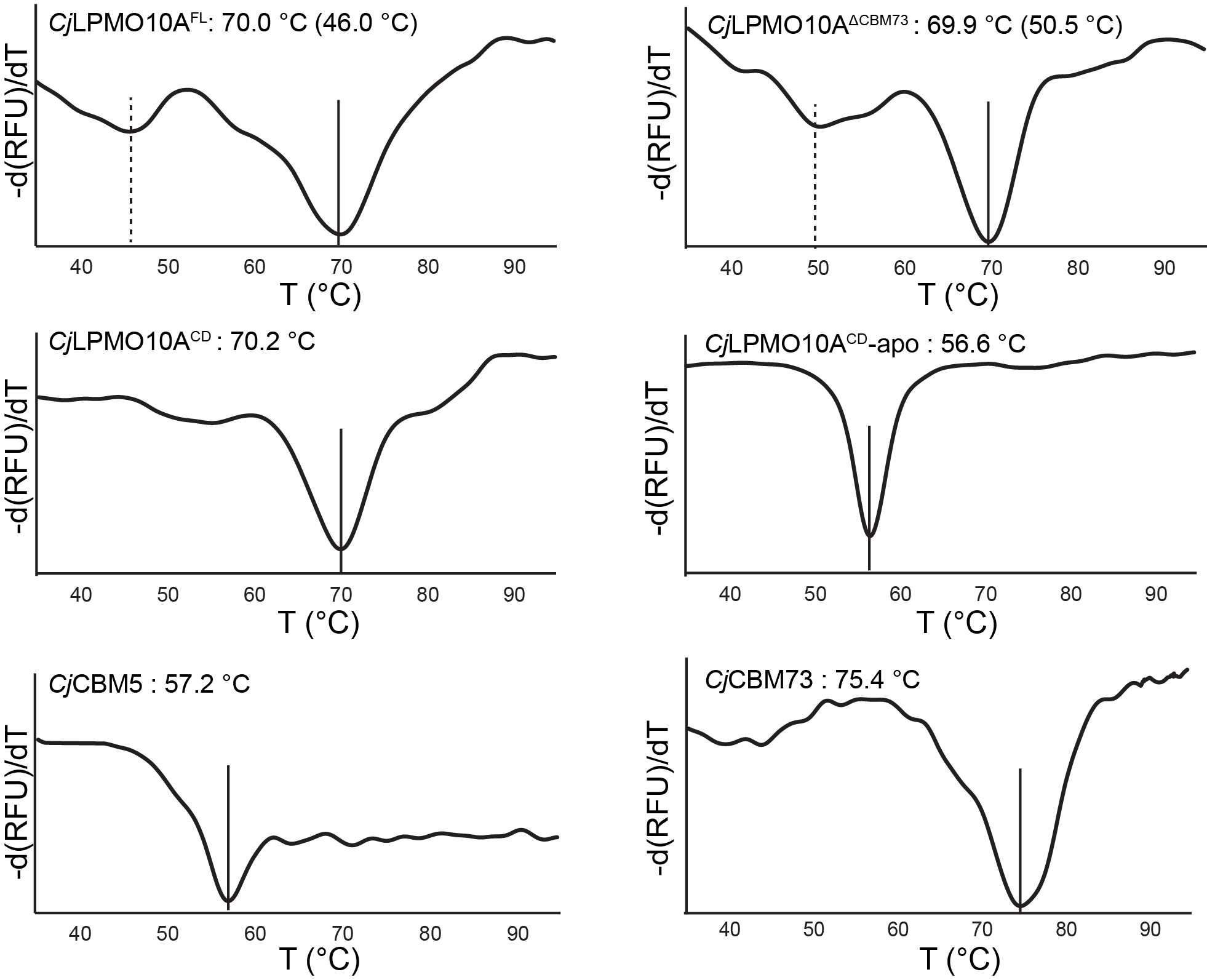
**

**Figure S1.** Thermal stability of *Cj*LPMO10A variants. The plots show the melting curves and apparent melting temperatures (T_m_) for copper-saturated *Cj*LPMO10A^FL^, *Cj*LPMO10A^ΔCBM73^, and *Cj*LPMO10A^CD^, as well as the apo form of *Cj*LPMO10A^CD^, *Cj*CBM5 and *Cj*CBM73. The derivative of the fluorescence signal (-dRFU/dT, where “RFU” stands for relative fluorescence units”) is plotted as a function of the temperature (1). The reactions contained 0.1 g/L protein and were heated from 25 °C to 95 °C, at a rate of 1.5 °C /min, in the presence of SYPRO orange (a fluorescent dye). The scans were performed four times for each protein and the Figures show a typical scan. All apparent melting temperatures (T_m_) had standard deviations below ±0.3 °C.


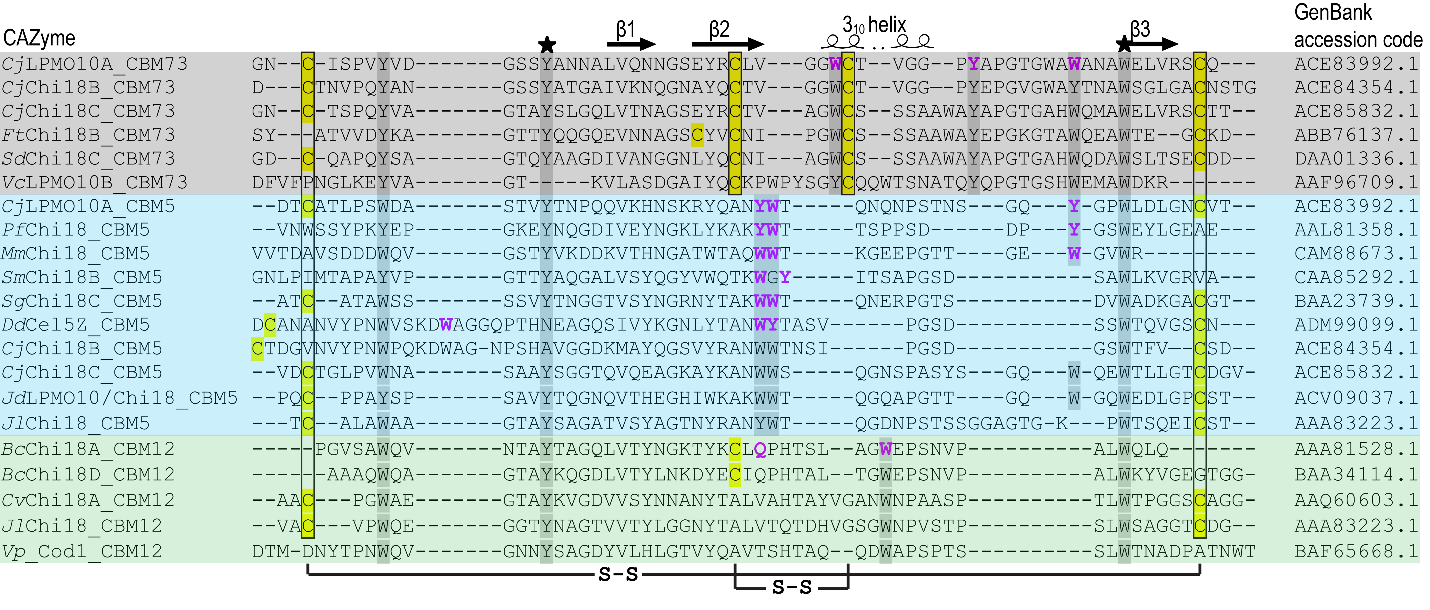
**Figure S2.**  Multiple sequence alignment performed on a selection of members of CBM5 (blue), CBM12 (green) and CBM73 (grey). The sequences are identified by the GenBank accession code and the CBM family registered in the CAZy database (2). The source organisms are abbreviated as *Cellvibrio japonicus* (*Cj*), *Francisella tularensis* (*Ft*), *Saccharophagus degradans* (*Sd*), *Vibrio cholerae* (*Vc*), *Pyrococcus furiosus* (*Pf*), *Mortella marina* (*Mm*), *Serratia marcescens* (*Sm*), *Streptomyces griseus* (*Sg*), *Dickeya dadantii* (*Dd*), *Jonesia denitrificans* (*Jd*), , *Janthinobacterium lividum* (*Jl*), *Bacillus circulans* (*Bc*), *Chromobacterium violaceum* (*Cv*), and *Vibrio parahaemolyticus* (*Vp*). Amino acids on the binding surface are highlighted in purple and correspond to the residues highlighted in Figure 8. These and other conserved aromatic residues are marked with grey boxes. The asterisks indicate two aromatic residues highly conserved in these CBMs, all buried in the hydrophobic core of these proteins. Conserved cysteines are marked with yellow boxes. The MSA was prepared using Clustal Omega 1.2.4 (3).


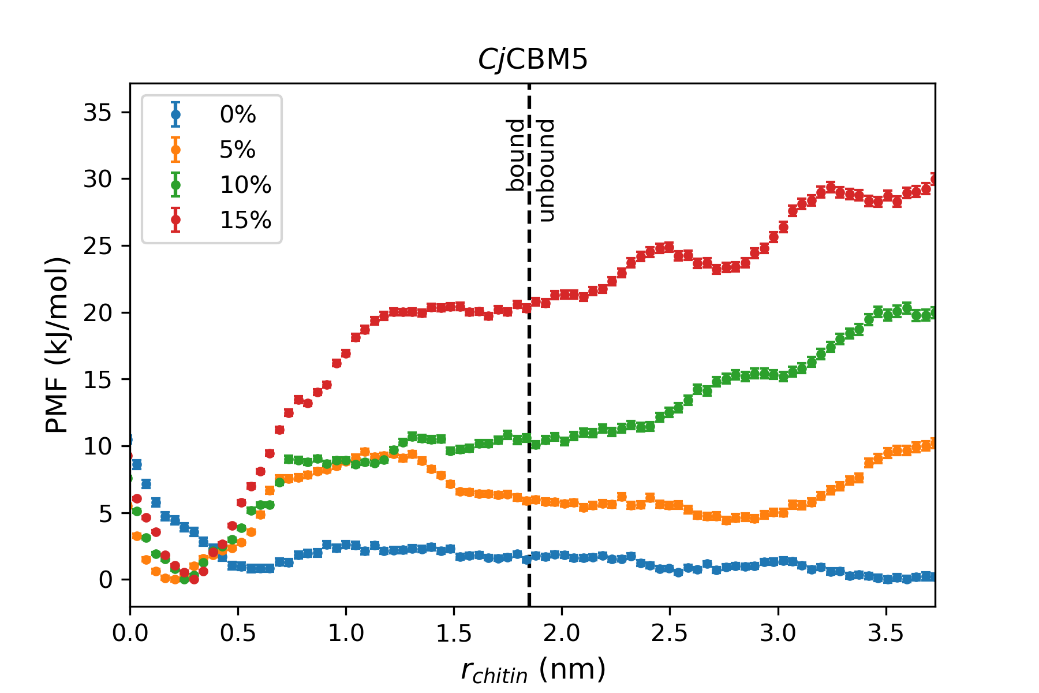


**
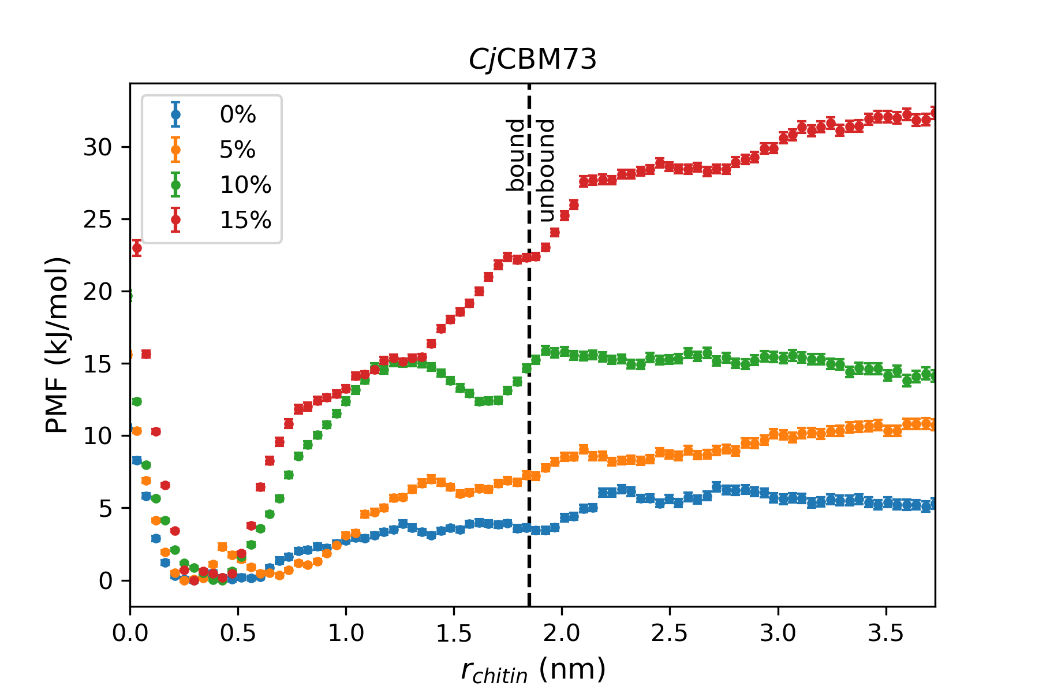
**

**Figure S3.** Potential of mean force (PMF) profiles for *Cj*CBM5 (top) and *Cj*CBM73 (bottom) as a function of their distance to the modeled α-chitin surface. The free-energy surfaces were calculated from umbrella sampling simulations performed at different chitin-protein interaction strengths: 0% (unchanged), 5% increase, 10% increase and 15% increase, and analyzed using the weighted-histogram analysis method (4, 5). The collective variable (*r*_chitin_) is the Euclidean distance between the z-coordinate of the geometric center of the beads belonging to aromatic amino acids on the putative binding surfaces (*Cj*CBM5: Y282, W283, Y296; *Cj*CBM73: W371, Y378, W386) and the *z*-coordinate of the geometric center of all chitin beads. The cut-off value for between the bound and unbound states, *r*_c_ = 1.85 nm, is indicated with a dashed line. Dissociation constants ($K_{d}$) were calculated from the PMF profiles by using Equations 5–7. Note that for *Cj*CBM5, at 5% and 10%, the PMF profiles are similar from *r*_chitin_ = 0 nm up to *r*_chitin_ ≈ 1.4 nm, but with increasing *r*_chitin_, the 10% curve becomes steeper, whereas the 5% curve (on average) levels off. Therefore, integration of these curves reveals that a 10% increase results in stronger chitin-protein interactions than a 5% increase (Table 1).


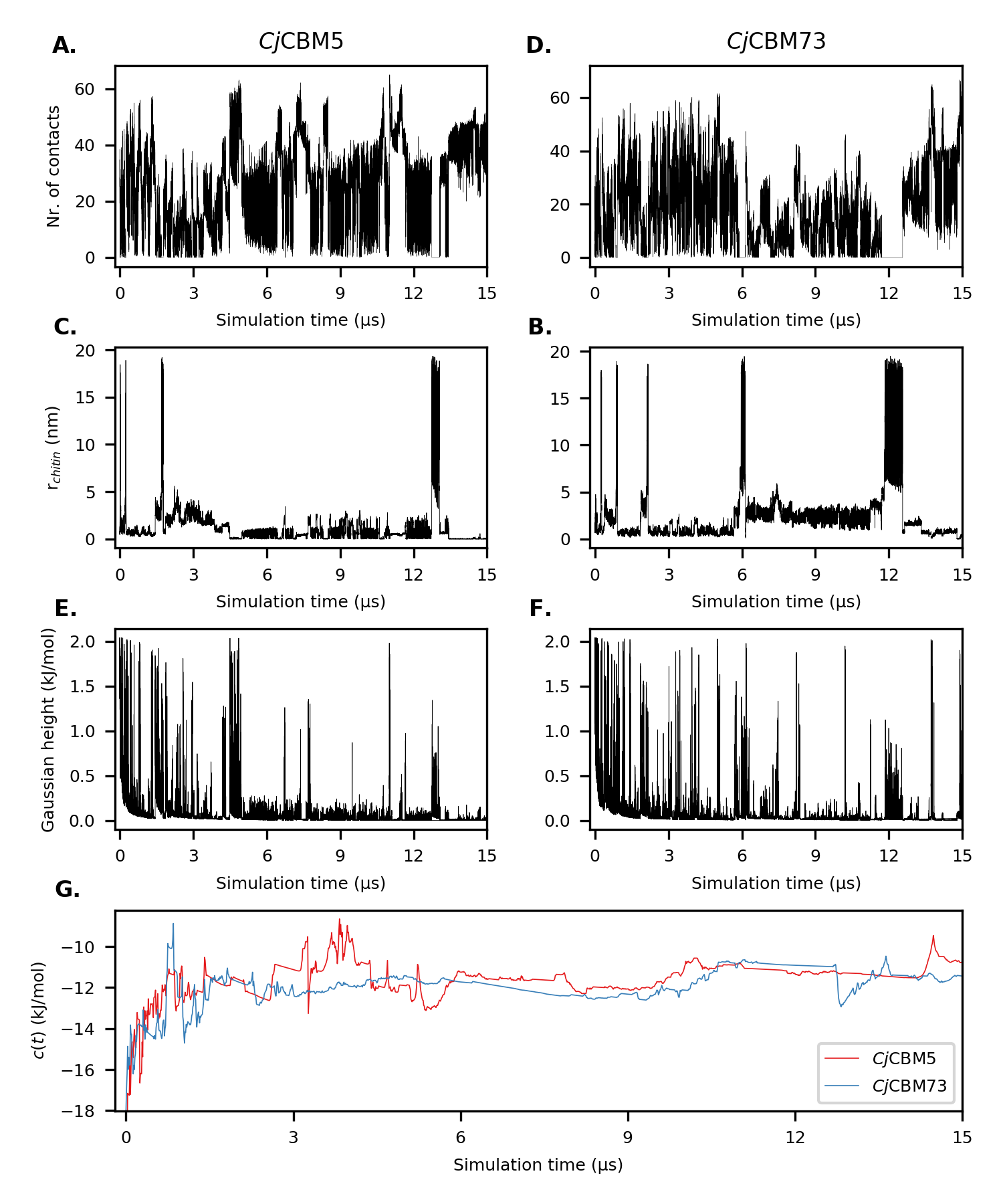


**Figure S4.** Time series of the number of contacts between the modeled α-chitin surface and selected amino acids on the surface of (A) *Cj*CBM5 (Y282, W283, Y296) and (B) *Cj*CBM73 (W371, Y378, W386), (C,D) the distance between these amino acids and the α-chitin surface (*r_chitin_*), and (E,F) Gaussian hill deposition over the course of the WT-MetaD simulations. Panel G shows c(t), an estimate of the reversible work performed on the system at time t (6), calculated using https://github.com/lud0/comp-bio-tools/blob/master/reweight.py. The small number of transitions observed between the bound and unbound states of the CBMs, indicates that these simulations are not converged after 15 µs. However, for *Cj*CBM5, both experiments and simulations find amino acids in the same region of the protein to be involved in (GlcNAc)_6_ and α-chitin contacts, respectively (Figure 7).

**References**

1. Huynh, K., and Partch, C. L. (2015) Analysis of protein stability and ligand interactions by thermal shift assay. *Curr. Protoc. Protein Sci.* **79**, 28.9.1-28.9.14

2. Levasseur, A., Drula, E., Lombard, V., Coutinho, P. M., and Henrissat, B. (2013) Expansion of the enzymatic repertoire of the CAZy database to integrate auxiliary redox enzymes. *Biotechnol. Biofuels*. **6**, 41

3. Sievers, F., Wilm, A., Dineen, D., Gibson, T. J., Karplus, K., Li, W., Lopez, R., McWilliam, H., Remmert, M., Söding, J., Thompson, J. D., and Higgins, D. G. (2011) Fast, scalable generation of high-quality protein multiple sequence alignments using Clustal Omega. *Mol. Syst. Biol.* **7**, 539

4. [online] Grossfield, A. (2020) WHAM: the weighted histogram analysis method, version 2.0.9. http://membrane.urmc.rochester.edu/?page_id=126 (Accessed April 14, 2021)

5. Kumar, S., Rosenberg, J. M., Bouzida, D., Swendsen, R. H., and Kollman, P. A. (1992) The weighted histogram analysis method for free-energy calculations on biomolecules. I. The method. *J. Comput. Chem.* **13**, 1011–1021

6. Tiwary, P., and Parrinello, M. (2015) A time-independent free energy estimator for metadynamics. *J. Phys. Chem. B*. **119**, 736–742
